# Supplementary figures and images for: Assessing the predictive value of morphological traits on primary lifestyle of birds through the extreme gradient boosting algorithm
Source: PLoS One. 2024 Jan 5;19(1):e0295182. doi: 10.1371/journal.pone.0295182 (PMC10769058; doi:10.1371/journal.pone.0295182)

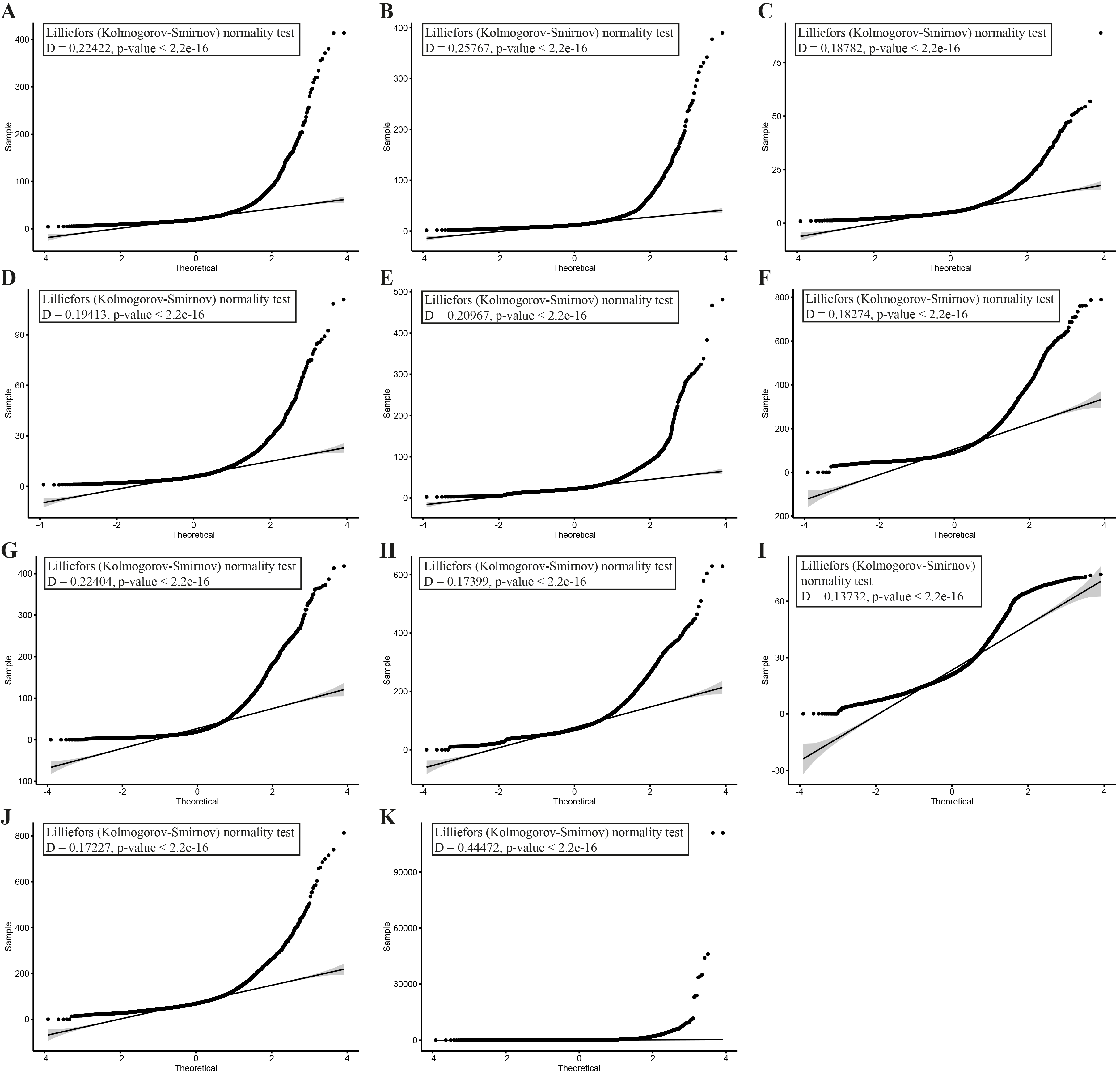

Supplement: S1 Fig — Each plot has the corresponding results of the Lilliefors (Kolmogorov-Smirnov) test of normality, which are the statistic D and the probability value associated. A. Beak length measured from tip to skull along the culmen (BLC). B. Beak length measured from the tip to the anterior edge of the nares (BLN). C. Beak width. D. Beak depth. E. Tarsus Length. F. Wing length from carpal joint to wingtip measured on the unflattened wing. G. Kipp’s distance H. Secondary length from carpal joint to tip of the outermost secondary (SL). I. Hand-Wing index J. Tail length. K. Mass. (TIF) [file pone.0295182.s001.tif]
