# Supplementary material for: Assessing the predictive value of morphological traits on primary lifestyle of birds through the extreme gradient boosting algorithm
Source: PLoS One. 2024 Jan 5;19(1):e0295182. doi: 10.1371/journal.pone.0295182 (PMC10769058; doi:10.1371/journal.pone.0295182)
Supplement: Attachment — Submitted filename: commentsToManuscript.pdf [file pone.0295182.s002.pdf]

Comments to “Assessing the predictive value of morphological traits on primary lifestyle of birds through the extreme gradient boosting algorithm”

GENERAL COMMENTS

Dear author, I found the topic of your research very interesting but also encountered important structural problems. I did not comment on the entirety of the manuscript as I think that most of my comments apply to all the sections in the text. In general I would identify the main weakness of this work in the structure of the text, which is often convoluted and hides the main messages/results of the study. A lot of methodological details are presented outside of the methods section and with very specific terms and confuse the reader of what is the main point of each section. Each section of a manuscript has a specific role in telling the story and by respecting these roles we ensure that the reader can follow the story and look for the sections he is mostly interested in. I tried to suggest some general rules that I hope can guide the author in future versions of the manuscript. My general suggestion is to simplify the text, reduce the jargon and follow the formal structure suggested in scientific writing to make the readers’ task a bit easier and help them as much as we can in disentangling our complex analyses. Our main aim as writers here should be to make sure that the reader can get out of our manuscript as much information as possible in very short time.

MINOR COMMENTS

- In the first page next to the author name it says “Bachelor of Biology”, I would discourage from writing your title next to your name to minimise bias
- at least two different fonts throughout the text, and also in general the font used by the author makes the text hard to read.

DETAILED COMMENTS

ABSTRACT:

- The abstract is very long with numerical information that are not needed here. The abstract should be the summary of our story, with a background, a gap of knowledge and a very short summary of the methods and results/take home messages. At lines 29 to 35 there are methodological details that belongs to the methods section not to the abstract. Background and gap of knowledge are missing (especially gap of knowledge expected after line 20, e.g. if many studies already did it, then how is this different from previous studies?). Lines 36 to 43: these results are too specific for the abstract section, what the reader expects here is general results written in jargon-free language and the take-home message for the study. The conclusion at line 43 says “This is another step forward in our mechanistic understanding of bird ecology.” but I think that the conclusion of the study should be elaborated on more than that.

INTRODUCTION

- l 45, what is an angular stone?
- l 47 increasing interest
- l 47-52. Here there is need for a more general introduction, this is too detailed for the broad readership of this journal. A reader that did not study ecology (and even one who did) might find this introduction confusing and too detailed. Start with a few lines of general introduction such as how is a trait defined, difference between trait and functional trait, why are traits important, why are they used in ecological studies, what are the lifestyles that you want to relate the traits to.
- l 52 I am confused about what is an ecological system (a species, a population, a community). And how do we transition from talking about ecological system in general to birds in specific? (l 53)
- l 54 what about reproduction?
- l 59 says “narrow wings perform best for gliding and soaring” I am not sure this is the case. See e.g. work from Pennycuick
- l 61 I could not follow why a lower metabolic rate limits dispersion ability. There are very large migrating birds who can travel very long distance, and I am not sure why would they have lower

dispersion ability. Also dispersion can be of seeds, of microorganisms, of diseases, etc; depending on what is being dispersed, different traits would come into play.
- l 66 to 82 these paragraphs have a lot of jargon (technical language), and by the end of the introduction the reader cannot understand why this study is needed and what the purpose of the study is, which is the main point that the introduction section should fullfill. I would recommend the author to revise the manuscript following general guidelines regarding the structure of a scientific manuscript.

- l 97 a definition of what these lifestyles are is recommended. Ideally you would not want the reader to read another paper before being able to understand yours; on the opposite the author wants to make the reader's job as easy as possible to make sure they take home the most they can from the paper.

DISCUSSION
- The first paragraph of the discussion should be rewritten, a discussion starts with a summary of what the results of the study are and then slowly puts them in context and finally highlights the relevance of the results for the field, from specific to general, somehow opposite to the introduction.
- l 309-310 says: "Univariate comparisons between levels in the response variable according to each predictor showed some insights that probably were exploited by the extreme gradient boosting algorithm." this sentence is not providing the reader with any information.
- l 305-310 this belongs more to the methods than the discussion, too many methodological details here.
- I 310-315 this seems a summary of the results (the one that should go in the first paragraph of the discussion) but it is somehow cryptic, it needs some clarification for readers that are not familiar with traits and with PCA or the specific methods. The discussion should be understandable for any reader especially as this journal has a wide readership. As a suggestion, when you write this, try to think that you would like to explain it in words to a colleague from a different field, and after it is clear and easy to read you can polish it.
- paragraph at l 318: here it is the same, too complex and a lot of methodological details that are telling me how the result was obtained but not really what the result was.. Ideally a reader should be able to get an idea of what a study found just by reading the abstract and the discussion. You can try this as an exercise on your own, by reading only the abstract and the discussion, putting yourself in the shoes of a naive reader, and seeing if the message is still clear when you omit methods etc.
- paragraph at l 325: this is interesting information from other studies, but now how your study fits in this picture is missing. In the discussion the author first summarises the results, and then they pick up on some, or all, of them and place them in the context of what other researchers did, and this can also be applied to the methods, especially if you think that a specific method can justify the difference between your results and results from other studies.
- The conclusions flow well and provide the reader with what they are expecting as a final take home message.
